# Pharmacological cognitive enhancement and the value of achievements: An intervention

**DOI:** 10.1111/bioe.13107

**Published:** 2022-11-18

**Authors:** Emma C. Gordon, Rebecca J. Willis

**Affiliations:** ^1^ Department of Philosophy University of Glasgow Glasgow UK; ^2^ BPP Law School BPP University London UK

**Keywords:** cognitive enhancement, ethics of enhancement, human enhancement, value of achievement

## Abstract

Pharmacological cognitive enhancements nontherapeutically improve cognitive functioning, though recent critics have challenged their use by claiming that cognitive success, aided by the use of cognitive enhancement, is less valuable than otherwise. We criticize two recent responses to this objection, due to Carter and Pritchard and Wang, and propose a different response on behalf of proponents of cognitive enhancement that is shown to be more promising.

## 1 INTRODUCTION

A thriving area of recent research in bioethics considers both the capability and desirability of medicine and technology to improve human functioning across various dimensions (including moral^1^, emotional,^2^ and cognitive).^3^ For instance, a number of bioconservative researchers worry that “enhanced” capabilities will not so straightforwardly contribute to better lives^4^ and that our optimism should be at best tempered by a sober assessment of the risks new enhancement technologies bring.

In this paper, we are interested in one particular form of enhancement—*cognitive enhancement*—and one particular objection—the *axiological objection—*that several recent papers use to challenge the use of such enhancement.^5^ By “cognitive enhancement,” we refer here to a broad category of pharmaceutical drugs that nontherapeutically improve our cognitive abilities.^6^ For example, modafinil is often used by students to increase wakefulness and concentration^7^, and we are likely to have similar, more effective drugs in the future.^8^

The *axiological objection* to cognitive enhancement draws on the widespread pretheoretical intuition that when an agent relies on something external to their agency and efforts (e.g., drugs or technology) to achieve a given cognitive aim, the resulting cognitive achievement (e.g., knowledge, understanding, etc.) is *less valuable* than it would be otherwise. The core thought is that this threat to “undercut” the value of our successes gives us *pro tanto* reason to refrain from using or pursuing research into such enhancements.^9^

In what follows, we argue that the best line of defense for proponents of cognitive enhancement against this objection type requires a distinction between (i) *immediate* success attained through a combination of one's ability and a given cognitive enhancement and (ii) potential *future* successes for which present enhancements function as enabling conditions.

In section one, we outline the axiological objection in more detail. In section two, we explain and evaluate response strategies from Carter and Pritchard^10^ and Wang.^11^ We then propose our limited defense of cognitive enhancement against the axiological objection. The result, we think, is a new way for proponents of cognitive enhancement to address the core of the objection while at the same time accommodating some of the underlying intuitions that account for its persuasiveness.

## 2 UNPACKING THE AXIOLOGICAL OBJECTION

Let us begin by taking a closer look at the axiological objection to cognitive enhancement, that is, roughly, the claim that cognitive enhancement reduces the value of achievements. Illustrative examples often revolve around enhancements removing certain *obstacles* to achieving cognitive tasks, making the intellectual process comparatively easier. Familiar obstacles in the cognitive domain include distraction,^12^ poor memory,^13^ fatigue, and frustration,^14^ for example. We can find the axiological objection expressed in slightly different ways throughout the bioconservative literature, including in work by Kass, Sandel, and Harris.^15^ Kass articulates the objection in terms of cognitive enhancements undermining the value of achievement by severing the link between performance and *effort*, thus making enhanced successes too “easy” to be of substantive worth.^16^ Similarly, Sandel argues that cognitive enhancement undermines achievements by disconnecting success from our agency where credit goes not to the enhanced subject so much as “to the pharmacist.”^17^ Harris, by contrast, claims that since the value of our achievements is predicated upon the possibility of failure, reducing or removing that freedom concurrently reduces the value.^18^

Carter and Pritchard use the example of a “cheat code” to illustrate the idea at the heart of all of these strands of the axiological objection.^19^ To see how this analogy is supposed to work, imagine a cheat code to a computer game that removes obstacles in such a way as to make winning very easy or almost guaranteed. The success of winning the game under such conditions is intuitively not (nearly) as valuable as it would be otherwise. To the extent that cognitive enhancements (viz., cognition‐boosting pharmacological enhancements, but potentially also technological intelligence augmentation)^20^ reduce our successes manifesting the effortful exercise of ability, these successes then become increasingly akin, axiologically, to a game played with salient obstacles removed.

Carter and Pritchard's distinction between *strong* and *weak cognitive achievements* offers further terminology for sharpening the formulation of the above objection. As they characterize it, a *weak achievement* is merely a success due to ability, for example, rather than luck.^21^ For example, if I intentionally lift a cup off the table, that is a success due to a very basic ability I possess. In contrast, a *strong achievement*—which Carter and Pritchard and others such as Bradford^22^ maintain is more valuable—is a success from ability (viz., a weak achievement) that, *additionally*, involves either or both (i) the overcoming of a significant obstacle or (ii) the exercise of a significant skill. An example of overcoming a significant obstacle might be completing a marathon despite physical fatigue; an example of exercising a significant skill might be a top footballer scoring a goal against a strong opposing team. If we import this terminology into discussions of the axiological objection, we have a further way to describe what Kass, Sandel et al. are worried about, namely, that cognitive enhancement tends to leave us with, at most, a life of less valuable, “weak achievements” when we may have otherwise been in the market for strong achievements.

## 3 RESPONSES TO THE AXIOLOGICAL OBJECTION: CARTER AND PRITCHARD (2019) AND WANG (2021)

In defending cognitive enhancement against the axiological objection, Carter and Pritchard invite us to consider “Moddy,” a student who uses modafinil when completing a maths puzzle. If she did not use it, suppose, she would have given up from exhaustion, lack of focus, or frustration. They argue, and we do not dispute, that Moddy's completion of the maths puzzle might still constitute a case of *strong* cognitive achievement, as Moddy's success might *still* (despite the use of modafinil) be primarily due to her (significant) mathematical skills. To make the example more concrete, suppose that the maths puzzle Moddy is engaged with is Fermat's Last Theorem and that (like Andrew Wiles, suppose) Moddy has been working at this for over a decade, integrating multiple branches of mathematics in an effort to approach a viable proof. However, we may add to the story, during the final stretch, Moddy's focus and effort begin waning. Stipulate that *without modafinil* Moddy would have fallen just short of proving the theorem; due to the modafinil, she stays the course and proves it.

Fleshed out with these details, we can see how the successful completion of a cognitive task might be such that (i) it depends *indispensably* on the use of an enhancer (such that, without the drug, the success would not have been achieved) and (ii) at the same time, be creditable to a significant level of skill. Cases like this, Carter and Pritchard maintain, cast doubt on the scope of the axiological objection. They reveal a compatibility between enhancement use and creditability to (significant) skill.

Wang, however, holds that the above line of argument does not satisfactorily address the extent to which ready access to cognitive enhancements will often enough (even if not always) undermine the value of achievement by artificially offsetting the kind of *difficulty* that contributes significantly to the value of a given achievement. Wang's line, accordingly, draws from Bradford's^23^ view that difficulty (and corresponding exertion of the will to overcome difficulty) best explains why we value achievements over *mere* successes.

As Wang sees it, the best response to the axiological objection is to point to how we might *manifest* virtue in a valuable way *in our use* of cognitive enhancements.^24^ For example, we can envision more and less responsible ways of incorporating cognitive enhancements into our cognitive architecture, ways that draw upon other intellectual virtues. Over time, Wang thinks, we might virtuously *integrate* enhancements into our own agency^25^ through extended responsible use.

Both Carter and Pritchard's and Wang's responses have important limitations. In the former case, the worry is that simply granting that enhancement is compatible with strong achievement is compatible with enhancement use *ordinarily enough* undermining the value of our achievements. Put another way, the proponent of the axiological objection can still claim (in response to Carter and Pritchard) that cognitive enhancement *usually* or typically lessens (even if not *always* so) the value of enhanced achievements, which is a significant concession.

The limitation of Wang's proposal is different. Even if we grant Wang that navigating our use of cognitive enhancements by drawing from our other intellectual virtues is valuable in its own right, the proponent of the axiological objection may point out that it remains that the enhanced successes *themselves* will be (qua enhancement dependent) of a lesser value, *unless*, for Wang, one (over time) “integrates” these enhancements into one's cognitive character. The problem at this point is that as the literature on cognitive integration suggests, “integrating” any kind of enhancement (pharmacological or otherwise) requires meeting a relatively high bar: either (i) the source of the enhancement's reliability must be reasonably well understood^26^ and/or (ii) the enhancement must (with reference to the dynamical systems theory)^27^ in some way generate “feedback loops,” that is, ongoing, two‐way causal interactions between the subject and the enhancement.^28^ While these conditions might sometimes be met in the case of technologically mediated enhancements (e.g., memory‐assisting technologies),^29^ we may expect that they (and especially the second condition) will be met less regularly in the case of pharmacological cognitive enhancement. Thus, as the worry goes for Wang, the axiological objection resurfaces in most cases of enhancement, where cognitive integration conditions will not plausibly be met.

## 4 A NEW WAY FORWARD

In this section, we want to register, and then develop in more depth, an idea, briefly touched upon by Carter and Pritchard, which we think has considerably more promise.

The idea can be illustrated with reference to the case of Moddy. Moddy (with the assistance of modafinil) will plausibly be more inclined than otherwise to attempt *even more* difficult problems, the problems she would not attempt in the first place without the aid of enhancement. As Carter and Pritchard note in passing, the conditional probability that Moddy will exhibit more strong cognitive achievements beyond the present task is higher given her use of cognitive enhancement in the present task than otherwise.

They devote little space to this “conditional probability,” style response (focusing centrally on the response noted in the previous section). However, we want to suggest that a version of this response that goes beyond what Carter and Pritchard themselves have suggested offers a different, and, we think, better, perspective from which the axiological implications of cognitive enhancements on cognitive achievement can be assessed and the problem addressed.

To appreciate the promise of (a version of) what is above a simple conditional probability reply, let us begin by noting two key observations. First, proponents of the axiological objection tend to advert (when articulating the significance of achievement value lost by an enhancement) to *future patterns* of enhancement use, for example, patterns that would (as Sandel puts it) lead to an “easy life.” Second, the value of an achievement (and by extension a given pattern of achievements) can plausibly be undermined not only by reliance on enhancement but also by lack of *ambition*, where the ambition level is multidimensional.^30^

Let us say that an achievement's ambition level, relative to a subject, is at least going to be a function of the following: (i) how much skill the achievement demands (relative to the subject's own skill levels)^31^; (ii) how much effort it demands (regardless of the skill required)^32^; (iii) how many obstacles the achievement requires overcoming^33^ regardless of (i, ii), and, crucially, (iv) the extent to which that achievement, if attained, would *exceed in dimensions (i)–(iii) the subject's previous track record of successes*.

Regarding (iv): suppose an individual has thus far (in some given domain of endeavor) *typically* only pursued cognitive tasks that are well within their comfort zone and limits when it comes to the skill/effort/and obstacle ambition metrics (i)–(iii). The point of including (iv) along with the more typical (i)–(iii) as an achievement ambition metric is that an attempt at a given achievement might well be more ambitious, and accordingly more valuable, on account of *diverging* (especially when diverging significantly so) from the subject's previous track record with (i)–(iii). Even though climbing Everest is objectively hard, the achievement of climbing Everest might, *for a subject with an established track record of climbing mountains that require great skill (i), effort (ii) and with many obstacles (iii)*, not do as well by the lights of ambition metric (iv) than (for, say a less able climber) the attempt at a moderately difficult mountain, against a background track record that includes comparatively (relative to that attempt) much lower score metrics for *skill (i), effort (ii), and (iii) obstacles*. Put another way, the achievement of a novice climber summiting a medium‐level mountain might be a more ambitious achievement overall than an expert climber's summiting Everest.

What goes for mountain climbing goes, *mutatis mutandis*, for cognitive achievements; the ambition level of a given cognitive achievement is plausibly a function of not only ambition metrics (i–iii) but also (iv).

But once this point is appreciated, the simple conditional probability observation (i.e., that one is likely to pursue more valuable achievements predicated on enhancement use than otherwise) begins to carry more argumentative weight. The linking premise here that gets us a *bona fide* response to the axiological objection connects the use of enhancements with the ambition dimension (iv). To see this, consider again the Moddy case, and let us even grant that Moddy's getting the right result on the math problem pursued *depended* on her use of modafinil; suppose she would have lost focus otherwise. Compare this now with a variation on the case where we hold everything fixed *except* that Moddy did not use the enhancement and (thus) did not solve the problem.

Given what we are conceding to the proponent of the axiological objection, we will assume that Moddy's achievement is ceteris paribus *less valuable* than it would be were she to have attained the same end unenhanced. However, the situation shifts (due to the ambition dimension (iv)) when we ask about *future patterns* of enhancement use. Here, when comparing the original case with the ‘nonenhancement' version of the case, we can reliably predict future patterns that score higher on the ambition dimension (iv) in the former case.

Here, it is useful to note psychological research on ambition in goal‐setting,^34^ which indicates that, among other factors that explain the pursual of, and commitment to, increasingly more challenging goals (relative to one's previous track record) is the self‐confidence associated with present success.^35^ Put simply, the attainment of cognitive objectives, *even* if aided by cognitive enhancements, patterns with the more ambitious forward‐goal setting than otherwise, contributing to ever‐more ambitious downstream achievement attempts.

This core idea gains further support from work on drive theories of curiosity.^36^ On these views, intellectual goal setting in inquiry is partly driven by our *emotive* responses to the acquisition of new knowledge that conflicts with one's previous conception of a subject matter, creating “information gaps.” By facilitating knowledge acquisition, cognitive enhancements may also plausibly contribute to an increased sense of curiosity that *drives* further and more ambitious goal‐setting, aimed at closing new information gaps.^37^

Taken together, these points recommend a broader way of assessing our patterns of achievement, one that takes into account goal‐setting expectations, which suggests that at least one important dimension of the ambitiousness of achievement (i.e., the extent to which it diverges from past track records in other ambition dimensions) will very plausibly be driven up by the success that enhancement use facilitates. This is so *even if* the use of enhancement has a deleterious effect on the value of any given achievement with reference to *other* contributing factors to an achievement's value (e.g., the contribution of skill and/or the overcoming of obstacles).

Whether these other factors somehow “trump” what we have called dimension (iv) (which is facilitated by enhancements) remains an open question. However, crucially, unless proponents of the axiological objection offer a reason to think that dimension (iv) is trumped by these other factors, the axiological objection remains undermotivated and is thus in need of further defense.

## CONFLICT OF INTEREST

The author declares no conflict of interest.

## Footnotes

1 de Melo‐Martín, I. (2018). The trouble with moral enhancement. *Royal Institute of Philosophy Supplement*, *83*, 19–33, https://doi.org/10.1017/s1358246118000279; Harris, J. (2011). Moral enhancement and freedom. *Bioethics*, *25*(2), 102–111.

2 Wasserman, D., & Liao, S. M. (2008). Issues in the pharmacological induction of emotions. *Journal of Applied Philosophy*, *25*(3), 178–192.

3 Sandberg, A., & Bostrom, N. (2006). Converging cognitive enhancements. *Annals of the New York Academy of Sciences*, *1093*(1), 201–227; Gordon, E. C., & Dunn, L. (2021). Pharmacological cognitive enhancement and cheapened achievement: A new dilemma. *Neuroethics*, *14*(3), 409–421; Bostrom, N., & Sandberg, A. (2009). Cognitive enhancement: Methods, ethics, regulatory challenges. *Science and Engineering Ethics*, *15*(3), 311–341.

4 Harris, op. cit. note 1; Kass, L. (2002). *Life, liberty and the defense of dignity: The challenge for bioethics*. Encounter Books; Sandel, M. J. (2007). *The case against perfection: Ethics in the age of genetic engineering*. Harvard University Press.

5 Harris, op. cit. note 1; Kass, op. cit. note 4; Sandel, op. cit. note 4; Wang, J. (2021). Cognitive enhancement and the value of cognitive achievement. *Journal of Applied Philosophy*, *38*(1), 121–135; Forsberg, L. (2013). No pain, no gain? Objections to the use of cognitive enhancement on the basis of Its potential effects on the value of achievement. In E. Hildt & A. G. Franke (Eds.), *Cognitive enhancement: An interdisciplinary perspective* (pp. 159–171). Springer; Radoilska, L. (2010). An Aristotelian approach to cognitive enhancement. *The Journal of Value Inquiry*, *44*(3), 365–375. https://doi.org/10.1007/s10790-010-9233-1

6 Bostrom & Sandberg, op. cit. note 3.

7 Estrada, A., Kelly, A. M., Webb, C. M., Athy, J. R., & Crowley, J. S. (2012). Modafinil as a replacement for dextroamphetamine for sustaining alertness in military helicopter pilots. *Aviation, Space, and Environmental Medicine*, *83*(6), 556–567.

8 Gordon & Dunn, op. cit. note 3; Clark, A. (2003). *Natural‐born cyborgs: Minds, technologies, and the future of human intelligence*. Oxford University Press; Carter, J. A., & Pritchard, D. (2019). The epistemology of cognitive enhancement. *The Journal of Medicine and Philosophy: A Forum for Bioethics and Philosophy of Medicine*, *44*, 220–242.

9 Sandel, op. cit. note 4.

10 Carter & Pritchard, op. cit. note 8.

11 Wang, op. cit. note 5.

12 Accarino, A. M., Azpiroz, F., & Malagelada, J. R. (1997). Attention and distraction: Effects on gut perception. *Gastroenterology*, *113*(2), 415–422; Lavie, N. (2010). Attention, distraction, and cognitive control under load. *Current Directions in Psychological Science*, *19*(3), 143–148.

13 McDonald, A., Haslam, C., Yates, P., Gurr, B, Leeder, G., & Sayers, A. (2011). Google calendar: A new memory aid to compensate for prospective memory deficits following acquired brain injury. *Neuropsychological Rehabilitation*, *21*(6), 784–807; Sutton, J. (2006). Introduction: Memory, embodied cognition, and the extended mind. *Philosophical Psychology*, *19*(3), 281–289.

14 Nibbeling, N., Oudejans, R. R., Ubink, E. M., & Daanen, H. A. (2014). The effects of anxiety and exercise‐induced fatigue on shooting accuracy and cognitive performance in infantry soldiers. *Ergonomics*, *57*(9), 1366–1379.

15 Kass, op. cit. note 4; Sandel, op. cit. note 4; Harris, op. cit. note 1.

16 Bradford, G. (2013). The value of achievements. *Pacific Philosophical Quarterly*, *94*(2), 204–224.

17 Sandel, op. cit. note 4.

18 Harris, op. cit. note 1.

19 Carter and Pritchard, op. cit. note 8.

20 Palermos, S. O. (2014). Loops, constitution, and cognitive extension. *Cognitive Systems Research*, *27*, 25–41.

21 Pritchard, D. (2009). Knowledge, understanding and epistemic value. *Royal Institute of Philosophy Supplement*, *64*, 19–43. https://doi.org/10.1017/s1358246109000046; Greco, J. (2010). *Achieving knowledge: A virtue‐theoretic account of epistemic normativity*. Cambridge University Press; Greco, J. (2003). Knowledge as credit for true belief. In M. DePaul & L. Zagzebski (Eds.), *Intellectual virtue: Perspectives from ethics and epistemology* (pp. 111–134), Clarendon Press; Sosa, E. (2010). Value matters in epistemology. *The Journal of Philosophy*, *107*(4), 167–190; Carter, J. A., Jarvis, B. W., & Rubin, K. (2015). Varieties of cognitive achievement. *Philosophical Studies*, *172*(6), 1603–1623.

22 Bradford, op. cit. note 16; Bradford, G. (2015). *Achievement*. Oxford University Press.

23 Bradford, op. cit. note 16; Bradford, op. cit. note 21.

24 Wang, op. cit. note 5.

25 Pritchard, D. (2010). Cognitive ability and the extended cognition thesis. *Synthese*, *175*(1), 133–151. https://doi.org/10.1007/s11229-010-9738-y; Carter, J. A. (2018). Virtue epistemology and extended cognition. In H. Battaly (Ed.), *The Routledge handbook of virtue epistemology* (pp. 420–432). Routledge; Kelp, C. (2013). Extended cognition and robust virtue epistemology. *Erkenntnis*, *78*(2), 245–252; Palermos, op. cit. note 19.

26 Pritchard, op. cit. note 24; Greco, J. (2008). Cognitive integration and the ownership of belief. *Philosophy and Phenomenological Research*, *76*(1), 173–184. https://doi.org/10.1111/j.1933-1592.2007.00121.x; Palermos, S. O. (2014). Knowledge and cognitive integration. *Synthese*, *191*(8), 1931–1951; Carter, J. A., & Kallestrup, J. (2020). Varieties of cognitive integration. *Noûs*, *54*(4), 867–890.

27 Palermos, op. cit. note 19.

28 Palermos, op. cit. note 25.

29 Clark, op. cit note 8.

30 Hirschi, A., & Spurk, D. (2021). Striving for success: Towards a refined understanding and measurement of ambition. *Journal of Vocational Behavior*, *127*, 103577. https://doi.org/10.1016/j.jvb.2021.103577; Spenner, K. I., & Featherman, D. L. (1978). Achievement ambitions. *Annual Review of Sociology*, *4*(1), 373–420.

31 Carter & Pritchard, op. cit. note 8.

32 Bradford, op. cit. note 16.

33 Wang, op. cit. note 5; Carter & Pritchard, op. cit. note 8; Bradford, op. cit. note 21.

34 Hirschi & Spurk, op. cit. note 29; Spenner & Featherman, op. cit. note 29.

35 Poulsen, A. A., Ziviani, J., Kotaniemi, K., & Law, M. (2014). “I think i can”: Measuring confidence in goal pursuit. *British Journal of Occupational Therapy*, 77(2), 64–66.

36 Edelman, S. (2007). Curiosity and exploration. Retrieved from http://www.csun.edu/~vcpsy00h/students/explore.htm; Loewenstein, G. (1994). The psychology of curiosity: A review and reinterpretation. *Psychological Bulletin*, *116*(1), 75.

37 Edelman, op. cit. note 35.

